# Innovative 3D‐bioprinted microfibers in calcium phosphate cement platform with Nell‐1 to activate nerve‐bone axis for synergistic bone, vasculature, and nanofibrous nerve regeneration

**DOI:** 10.1002/smo2.70078

**Published:** 2026-07-15

**Authors:** Minjia Zhu, Xinyi Li, Jingyi Li, Kan Yu, Zixiang Dai, Le Xiao, Qinrou Zhang, Zihan Jia, Qingchen Qiao, Zeqing Zhao, Ke Zhang, Yuxing Bai

**Affiliations:** ^1^ Department of Orthodontics School of Stomatology Beijing Stomatological Hospital Capital Medical University Beijing China; ^2^ Department of Dentistry Beijing Friendship Hospital Capital Medical University Beijing China

**Keywords:** angiogenesis, bone tissue engineering, innervation, nanofibers, Nell‐1, osteogenesis, “nerve‐bone”axis

## Abstract

Critical‐sized bone defects remain a persistent clinical challenge, primarily because conventional scaffolds fail to reproduce the nanofibrous neurovascular architecture that underlies functional bone regeneration. Here, we present a breakthrough 3D‐bioprinted platform combining alginate microfibers (aMF) laden with human periodontal ligament stem cells (hPDLSCs) and a calcium phosphate cement (CPC) matrix, creating a hybrid environment that bridges mechanical stability with nanofibrous neurovascular guidance. This platform is engineered for the spatiotemporal delivery of Nell‐1, which uniquely activates a novel “nerve‐bone” axis. Nell‐1 engagement initiates a CYFIP1‐mediated CGRP‐β3 tubulin cascade that amplifies neurovascular and bone crosstalk. A dual‐phase release profile emerges as a critical design feature, with early signaling creating a favorable microenvironment for neurovascular infiltration, and then sustain signaling over time. Together, these phases synergistically enhance osteogenesis by a 2‐fold increase, angiogenesis by a 3‐fold increase, and innervation by a 2‐fold increase. When tested in rat cranial defects, our construct outperformed controls by doubling bone and nerve regeneration while tripling vascular density, thereby achieving unprecedented healing rates compared to existing approaches. Mechanistically, we redefine Nell‐1 as a dual osteo‐neurogenic regulator, leveraging endogenous stem cells to drive structural and functional repair. This 3D‐bioprinted microfiber‐in‐CPC system advances beyond passive scaffolds by dynamically coupling structural support with bioactive signaling, offering a transformative strategy for neuro‐vascularized bone reconstruction.

## Graphical abstract

A 3D‐bioprinted platform with stem cells activates “nerve‐bone” signaling by enabling the spatiotemporal delivery of Nell‐1. This activation enhances neurovascular and bone regeneration through the CYFIP1 cascade. In rat calvarial defects, the platform doubled bone regeneration and nerve repair and tripled vascular density, resulting in superior healing rates.

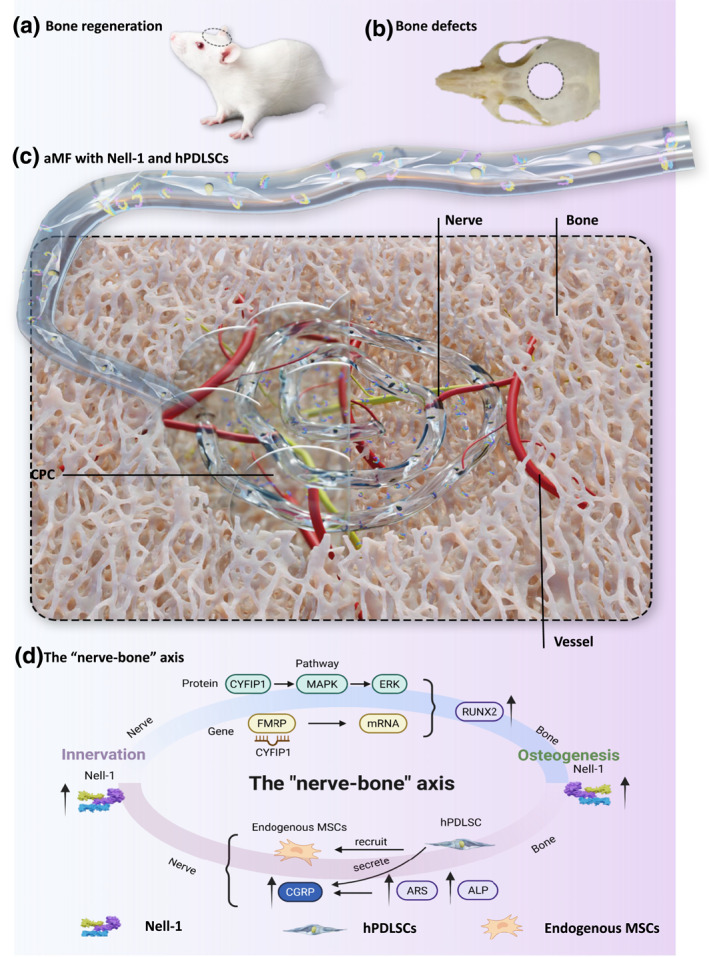

## 1 INTRODUCTION

The clinical management of critical‐sized bone defects resulting from trauma, oncological resection, or degenerative pathologies continues to present formidable challenges, with conventional bone tissue engineering strategies achieving only partial success.[^1^, ^2^, ^3^, ^4^] While current approaches have made significant strides in promoting osteogenesis, their predominant focus on mineralized tissue formation represents a fundamental limitation—the failure to recapitulate the essential neurovascular networks that underpin functional bone regeneration.[^5^, ^6^] Importantly, this oversight becomes particularly consequential in large, irregular defects where the natural healing cascade is compromised. Furthermore, emerging insights now reveal that complete bone regeneration is inherently a tri‐tissue process, requiring the synchronized development of osseous, vascular, and neural tissues. This process depends on dynamic crosstalk within the “nerve‐bone” axis, a critical neurovascular interface that coordinates bone healing and regeneration.[^7^, ^8^, ^9^, ^10^]

The concept of the “nerve‐bone” axis has emerged from accumulating evidence that skeletal tissue is not an inert structural framework but an active participant in neurobiological communication, as investigated by the latest research.^[11]^ Sensory, sympathetic, and parasympathetic fibers dynamically regulate bone metabolism through a combination of neurotransmitters such as calcitonin gene‐related peptide (CGRP), cytoskeletal markers including β3‐Tubulin, and bioelectrical signaling, while bone‐derived factors, most notably osteocalcin, feedback to influence neural development and plasticity via pathways involving Cytoplasmic FMR1 Interacting Protein 1 (CYFIP1).[^12^, ^13^, ^14^, ^15^] This neuro‐osseous cross‐regulation extends beyond mere structural coexistence; it forms the physiological foundation for coordinated tissue remodeling, mechanotransduction, and metabolic homeostasis. However, despite this well‐documented interdependence, the development of scaffolds capable of simultaneously supporting osteogenesis, angiogenesis, and innervation has remained an elusive goal in regenerative medicine. The challenge lies not only in recreating appropriate physical architecture but also in delivering the precise spatiotemporal sequence of biological cues required to activate this tripartite regeneration program. Our work addresses this fundamental limitation through the strategic application of Neural epidermal growth factor‐like 1 (Nell‐1), a uniquely pleiotropic factor with demonstrated efficacy in osteogenesis, angiogenesis, and neurogenesis.[^16^, ^17^] We present an engineered solution that combines advanced materials science with developmental biology principles, a hybrid scaffold system featuring dual‐phase release kinetics of Nell‐1 within a structurally and biologically optimized microenvironment.

Nanofibers process a controlled release characteristic.^[18]^ The scaffold design integrates two functionally complementary components: (1) a calcium phosphate cement (CPC) matrix providing immediate mechanical support and initial Nell‐1 release, and (2) 3D‐bioprinted alginate microfibers (aMF) encapsulating human periodontal ligament stem cells (hPDLSCs) for sustained trophic factor delivery.[^19^, ^20^] This configuration creates a precisely timed therapeutic sequence: the CPC‐embedded Nell‐1 generates an early osteo‐inductive and neurotrophic microenvironment (days 0–2), while the gradually degrading aMF release Nell‐1 during the critical window of neurovascular ingrowth (days 6–12), thereby spanning the entire regenerative timeline.

The selection of hPDLSCs as cellular components represents a deliberate strategic choice, capitalizing on their unique multipotency and neuro‐osteogenic paracrine activity.[^21^, ^22^] Unlike conventional MSC sources, hPDLSCs naturally reside in a neurovascular‐rich niche and demonstrate superior capacity for secreting factors that stimulate the “nerve‐bone” axis.^[23]^ When combined with our scaffold's architectural features, particularly the aMF‐generated microchannels that guide vascular and neural infiltration, this creates an unprecedented regenerative microenvironment that actively orchestrates all three tissue components.

What distinguishes our approach from previous attempts is its comprehensive treatment of bone regeneration as an integrated neurovascular‐osseous process rather than isolated tissue formation. The staged Nell‐1 delivery system represents the first demonstration of temporally programmed neuro‐osteogenic factor release, while the 3D‐bioprinted microfiber architecture provides both physical guidance and biological signaling in perfect spatial registration. Our results demonstrate that this dual‐phase system not only doubles osteogenic and neurogenic outcomes but also triples angiogenic activity compared to conventional scaffolds ‐ a quantitative leap in regenerative performance that directly addresses the clinical shortcomings of current technologies.

**FIGURE 1:**
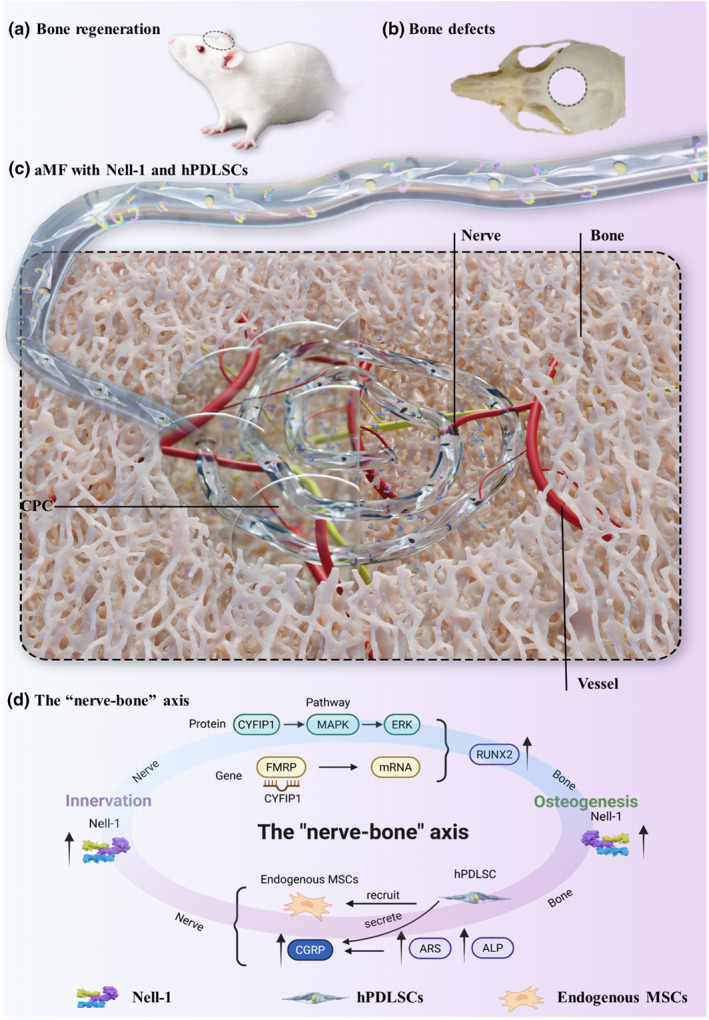
Schematic of study design. (a) 3D‐bioprinted aMF in calcium phosphate cement scaffold can repair large bone defects. (b) 3D‐bioprinted aMF in CPC scaffold can promote osteogenesis, angiogenesis, and innervation. (c) 3D‐bioprinted alginate hydrogel microfiber. The structures were 3D‐bioprinted with hydrogel, Nell‐1, and hPDLSCs as bioink. (d) Through the “nerve‐bone” axis, Nell‐1 could promote both osteogenesis and innervation.

Beyond its immediate therapeutic implications, this work establishes several conceptual advances: (1) it positions Nell‐1 as a master regulator of neuro‐osseous integration, (2) demonstrates that nerve nanofiber ingrowth is not merely a consequence but a requirement for optimal bone regeneration, and (3) provides a blueprint for next‐generation scaffolds that actively engineer multi‐tissue microenvironments through controlled spatiotemporal delivery. The successful integration of materials engineering, developmental biology, and clinical need in this platform represents a paradigm shift in bone tissue engineering ‐ from passive structural support to active microenvironmental instruction (Figure 1a–c).

This study aimed to: (1) develop a pioneering 3D‐bioprinted CPC‐aMF scaffold system featuring hPDLSCs encapsulation and programmed two‐stage Nell‐1 release and (2) systematically evaluate its capacity to drive coordinated osteogenesis, angiogenesis, and neurogenesis via the “nerve‐bone” axis in a rat cranial defect model‐a tri‐tissue regenerative approach previously unreported. We tested three hypotheses: (1) The 3D‐bioprinted aMF would maintain hPDLSCs viability while enabling controlled cell release and proliferation within CPCs porous architecture; (2) The scaffold's dual‐phase Nell‐1 delivery would synergize with hPDLSCs to achieve unprecedented tri‐tissue regeneration through neuro‐osseous crosstalk; (3) Nell‐1 would mediate bone‐nerve interactions via CYFIP1 upregulation, establishing a novel molecular pathway for neuro‐coupled bone regeneration. Through the “nerve‐bone” axis, Nell‐1 could promote both osteogenesis and innervation.

## 2 RESULTS

### 2.1 Effects of Nell‐1 on osteogenesis and its mechanism of hPDLSCs in vitro

**FIGURE 2:**
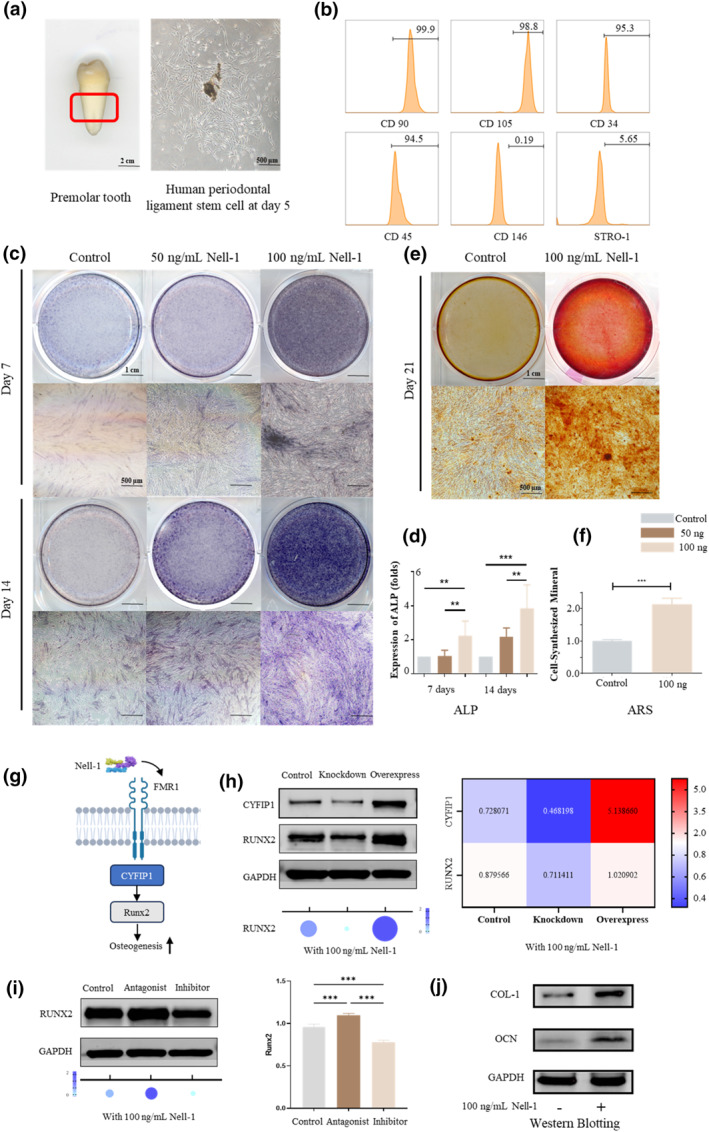
Osteogenic activity and its mechanism of hPDLSCs with Nell‐1 in vitro. (a) hPDLSC harvest and culture. (Scale bar = 2 cm and 500 μm) (b) Flow cytometry of hPDLSCs. (c) Alkaline phosphatase staining of hPDLSCs with 0 ng/mL, 50 ng/mL, and 100 ng/mL Nell‐1 after 7 and 14 days of culture. (Scale bar = 1 cm and 500 μm) (d) Osteogenic differentiation‐related genes (ALP) expression of hPDLSCs after 7 and 14 days of culture (*n* = 4). (e) An ARS staining of hPDLSCs with 0 ng/mL and 100 ng/mL Nell‐1 after 21 days of culture. (Scale bar = 1 cm and 500 μm) (f) Semi‐quantification of cell‐synthesized minerals of hPDLSCs with 0 ng/mL and 100 ng/mL Nell‐1 after 21 days of culture (*n* = 4). (g) Diagram illustrating the CYFIP1‐mediated cascade. (h) Western blot assays evaluating the expression levels of cytoplasmic FMR1 interacting protein 1 (CYFIP1) and Runx2 after knockdown and overexpression through shRNA (*n* = 4). (i) Western blot assays evaluating the expression levels of Runx2 after employing antagonist and inhibitor (*n* = 4). (j) Western blotting of osteogenic differentiation‐related genes (COL‐1, OCN) expression of hPDLSCs after 14 days of culture (*n* = 4). All values were presented as the mean ± SD, *p < 0.05, **p < 0.01, and ***p < 0.001 analyzed by *t*‐tests and one‐way ANOVA.

HPDLSCs, which are mesenchymal stem cells, can be easily harvested from human teeth^[24]^ and have been found to exhibit optimal osteogenic differentiation and could serve as multiple internal ossification centers in vivo (Figure 2a,b). Nell‐1 is an exocrine protein and a potent multifunctional bone growth factor. It was initially discovered in patients with unilateral coronal synostosis and has been found to play a crucial role in neural crest differentiation and regeneration.[^25^, ^26^] In this study, we aimed to investigate the effects of Nell‐1 on osteogenic, angiogenic, and innervation properties and its CYFIP1‐mediated cascade mechanism of hPDLSCs.

HPDLSCs were cultured with various concentrations of Nell‐1 for 7 and 14 days. Compared to 0 and 50 ng/mL Nell‐1 groups, hPDLSCs treated with 100 ng/mL Nell‐1 exhibited a significant 4‐fold increase in alkaline phosphatase (ALP) expression (Figure 2c) and semi‐quantification of ALP activity (Supporting Information S1: Figure S1A,B). The PCR results also showed a 4‐fold increase in ALP expression (Figure 2d). Additionally, higher concentrations of Nell‐1 (800 ng/mL and 1600 ng/mL) were also tested and showed similar ALP staining compared to the 100 ng/mL group (Supporting Information S1: Figure S1A). Further examination of osteogenic effects was conducted using alizarin red (ARS) staining and western blotting on day 21. Significant osteogenic effects were also observed in the 100 ng/mL group (Figure 2e,f,j).

To investigate the molecular mechanisms of osteogenesis, we conducted an RNA sequencing test.^[27]^ Among the identified enriched genes, CYFIP1 may play the most significant role in promoting innervation during bone reconstruction (Figure 2g). Transfection with shRNA plasmids was conducted to facilitate both the knockdown and overexpression of CYFIP1 within the Nell‐1‐induced “nerve‐bone” axis. Protein extraction and collection were performed. The results from the western blot analysis indicated that levels of CYFIP1 and Runx2 were significantly elevated in the overexpress group (Figure 2h). Moreover, CYFIP1‐cAMP agonist Forskolin and CYFIP1‐mTOR inhibitor rapamycin were also employed to specifically target CYFIP1. These results also confirmed that CYFIP1‐mediated cascade played an important role in Nell‐1‐promoted osteogenesis (Figure 2i).

Our study has, for the first time, revealed the dual regulatory role of Nell‐1 in osteogenesis via the “nerve‐bone” axis. The nerve‐derived pathway involves CYFIP1, which can upregulate genes related to osteogenesis and its corresponding protein Runx2. Moreover, the bone‐related growth factor Nell‐1 can enhance the expression and secretion of CGRP by hPDLSCs and other potential endogenous cells. Collectively, within our scaffold system, the processes of osteogenesis and innervation may synergistically activate the “nerve‐bone” axis, thereby promoting regeneration of vascular and nerve tissues along with bone.

### 2.2 RNA‐sequence profiling of Nell‐1 on hPDLSCs in vitro

**FIGURE 3:**
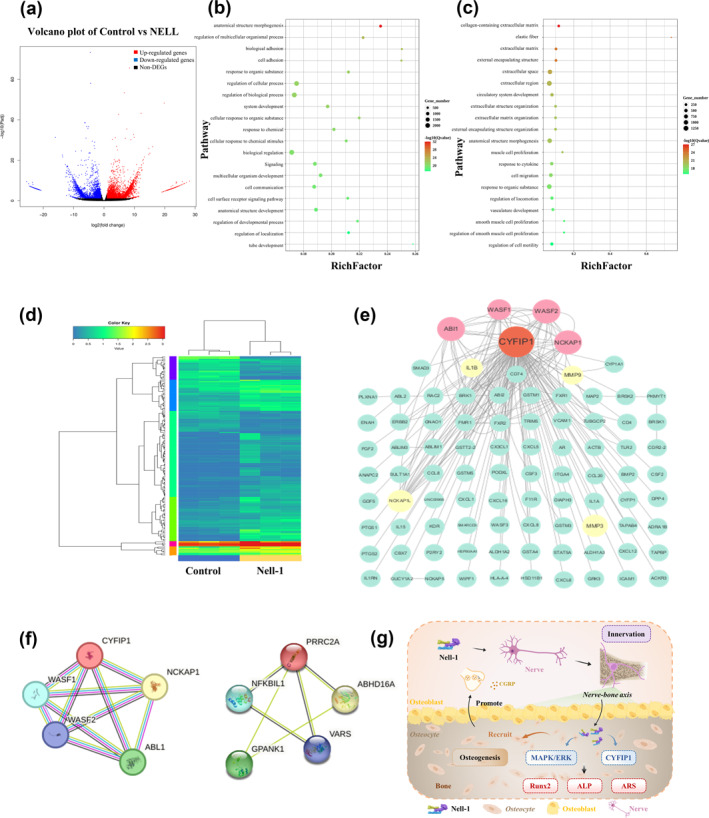
CYFIP1 and (Proline‐rich coiled‐coil 2A) PRRC2A pathway in hPDLSCs treated with Nell‐1. (a) A volcano plot of RNA‐seq results showing statistics of upregulated and downregulated genes. The up‐regulated, down‐regulated, and unchanged genes were dotted in red, blue, and black, respectively. (b, c) KEGG enrichment analysis for differentially expressed genes and proteins. (d) A cluster heatmap showed differentially expressed genes. (e) Up‐regulated protein‐protein interaction network of proteins. Nodes represent important proteins with complex social relationships. (f) A high‐scoring subnetwork containing CYFIP1, PRRC2A, and innervation. (g). Diagram illustrating the CYFIP1‐mediated nerve‐bone axis. CYFIP1, cytoplasmic FMR1 interacting protein 1.

To elucidate the mechanisms associated with neuro‐vascularization at a molecular level, we further conducted the functional gene profile and protein profile of hPDLSCs in both the negative control group and the 100 ng/mL Nell‐1 group through RNA sequencing. Analysis of the volcano plot revealed a total of 7348 genes that exhibited differential expression (log2FC > 1.0, *p* < 0.05). Among these genes, 4231 were found to be up‐regulated, while 3117 were down‐regulated, reflecting diverse expression patterns associated with ossification (Figure 3a).

Additionally, KEGG functional enrichment analysis was performed to identify numerous enriched biological pathways (Figure 3b,c). Upon further analysis, we were able to discern the top 20 signaling pathways associated with differentially expressed genes in relation to osteogenesis. Furthermore, GO term analysis also revealed distinct enriched genes that were promoted by Nell‐1 (Supporting Information S1: Figure S2). Moreover, we also evaluated the heatmap, which showcased diverse gene expression profiles when the growth factor Nell‐1 was introduced (Figure 3d).

Among the genes identified as enriched, CYFIP1 and proline‐rich coiled‐coil 2A (PRRC2A) appeared to play a particularly critical role in facilitating innervation throughout the process of bone regeneration (Figure 3e,f). Varying expression of CYFIP1 may lead to abnormalities in dendritic complexity, spine morphology, and synaptic function, which often lead to neuropsychiatric disorders.^[28]^ PRRC2A is crucial for m6A reading and has a strong connection with spermatogenesis,^[29]^ specialization, and myelination of oligodendrocytes.^[30]^ Further cluster analysis found that WASF1, WASF2, NCKAP1, and ABL1 may form a related network with CYFIP1, while NFKBIL1, GPANK1, ABHD16 A, and VARS form a related network with PRRC2A. Both pathways possess significant potential to contribute to nerve in‐growth during bone repair (Figure 3g).

### 2.3 Design and characterization of the 3D‐bioprinted aMF in CPC scaffold

3D‐bioprinted alginate hydrogel has been proven to be a reliable system for reproducing and repairing targeted tissues.^[31]^ To enhance the ability to modulate the bone regenerative environment and recruit endogenic growth factors, we innovatively developed a cutting‐edge controlled drug delivery system with multiple stages of growth factor release to activate the “nerve‐bone” axis. Specifically, we chose to encapsulate the growth factors in two parts of the scaffold: CPC and aMF (Figure 1c).

**FIGURE 4:**
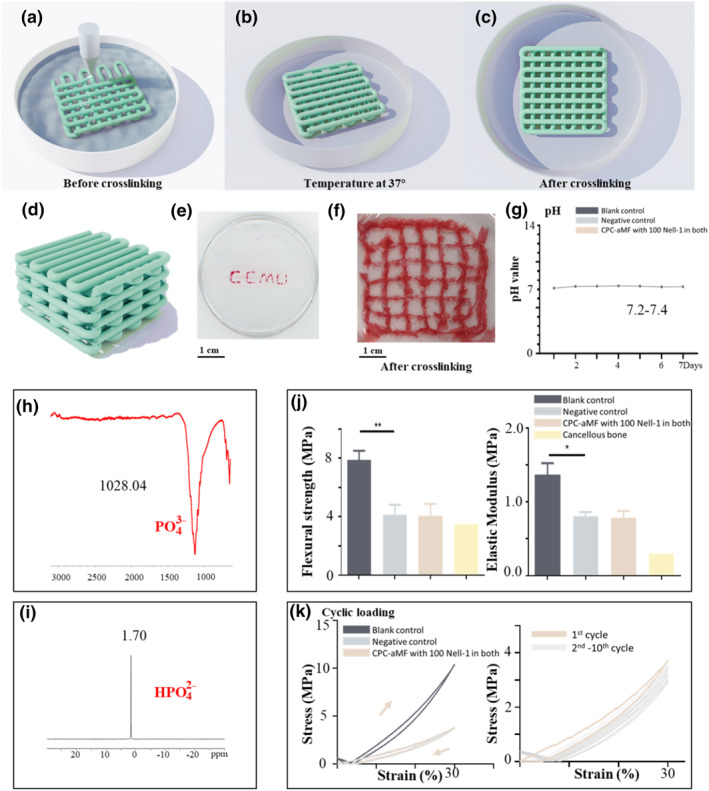
3D‐bioprinted aMF in CPC scaffold for large bone repair. (a–c) Schematic diagrams of the FRESH bath strategy for printing the hydrogel scaffold. Before crosslinking, heating to 37°C to melt the gelatin, and removing the suspension medium to finalize the crosslinking. (d) Computer‐aided 3D design model. (e) 3D‐printed letters: CCMU. (f) Gross appearance of the 3D‐printed scaffold. Scale bar = 1 cm. (g) pH changes of the scaffold. (h) Fourier‐transform infrared spectroscopy showing PO_4_^3‐^ in the CPC scaffold. (i) Atomic force microscope showing HPO_4_^2‐^ in the CPC scaffold. (j, k) Mechanical properties of the CPC‐microfiber scaffold: flexural strength, elastic Modulus, and cyclic loading. These groups were tested: Blank control (CPC scaffold only), Negative control (CPC‐aMF with 0 Nell‐1 control), CPC‐aMF scaffold with 100 ng/mL Nell‐1 in both (controlled two‐stage release of Nell‐1), cancellous bone (bone control), CPC‐aMF scaffold with 100 Nell‐1 in CPC, and CPC‐aMF scaffold with 100 Nell‐1 in MF. All values were presented as the mean ± SD, *p < 0.05, **p < 0.01, and ***p < 0.001 analyzed by one‐way ANOVA (*n* = 4). CPC, calcium phosphate cement.

**FIGURE 5:**
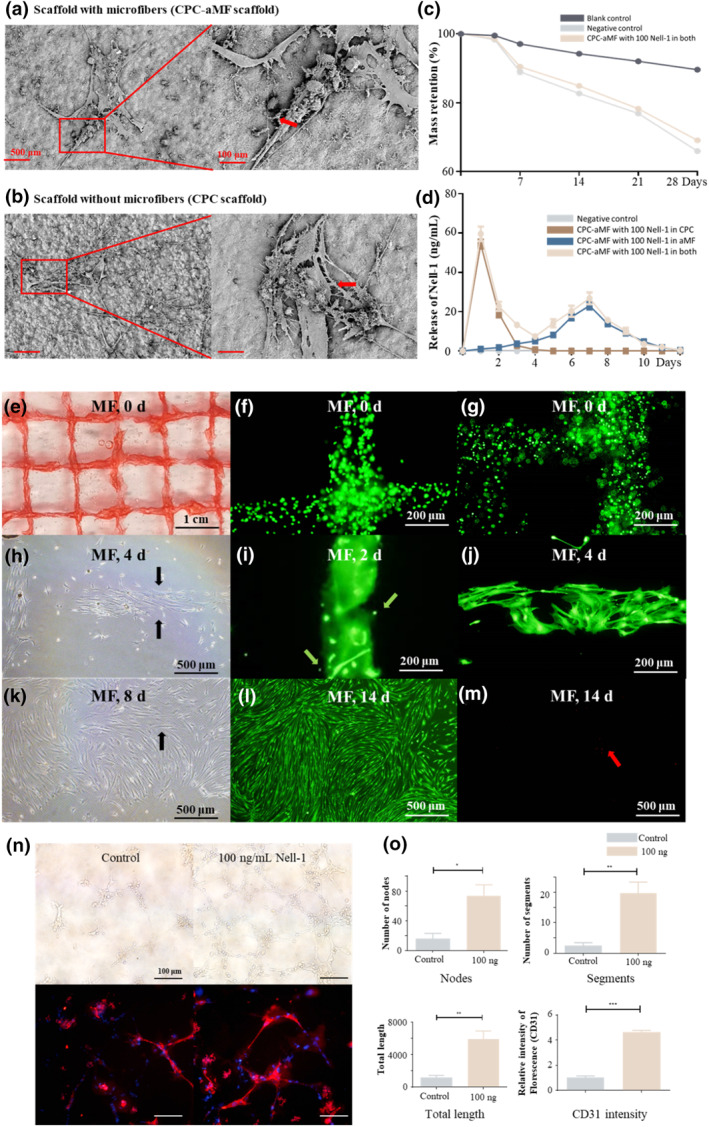
Characterization of 3D‐bioprinted aMF encapsulating hPDLSCs in CPC scaffold with Nell‐1. (a, b) Scanning electron microscopy (SEM) of the scaffold with and without microfibers. Scaffold with microfibers showed hPDLSCs stretching through pores after the degradation of microfiber (arrow). Scale bar = 500 μm (left) or scale bar = 50 μm (right). (c) In vitro long‐term degradation properties of CPC and CPC‐MF scaffold. (d) Two‐stage Nell‐1 releasing properties of the CPC‐microfiber scaffold. (e) Optical photographs of 3D‐bioprinted aMF in CPC scaffolds. Scale bar = 1 cm. (f), (g) Live/dead assay of the 3D‐bioprinted aMF in CPC scaffolds on day 0. Scale bar = 200 μm. (h, k) Microscopic appearance of the 3D‐bioprinted aMF in CPC scaffolds at days 4 and 8, showing the degradation of the microfiber (arrow) and proliferation of hPDLSCs. Scale bar = 500 μm. (i, j, l, m) Live/dead assay of the 3D‐bioprinted aMF in CPC scaffolds at 0, 2, 4, 8, and 14 days. The proliferation of hPDLSCs (green arrow as the live cells, and red arrow as the dead cells). Scale bar = 200 and 500 μm. (n) Tube formation of hPDLSCs with 0 ng/mL and 100 ng/mL Nell‐1. (Scale bar = 100 μm) (o) Quantitative analysis of the number of nodes, segments, and total length of the vascular networks. All values were presented as the mean ± SD, *p < 0.05, **p < 0.01, and ***p < 0.001 analyzed by *t*‐tests. CPC, calcium phosphate cement.

Seed cells, hPDLSCs, and the growth factor Nell‐1 were encapsulated in aMF (Supporting Information S1: Figure S3). The hPDLSCs and Nell‐1 were mixed in bioink, printed, and crosslinked with hydrogel using the FRESH protocol (Figure 4a–f, Movie S1). During the printing process, the needle diameter, speed, and extrusion pressure were maintained at a constant value of 27 G, 10 mm/s, and 1.5–1.8 kPa, respectively. The alginate microfiber and the spacing in‐between were approximately 100–300 μm (Figure 5e–g). Aligned diameter of the microfiber provides better oxygen permeability than a traditional single‐column alginate scaffold. CPC was synthesized based on our previous research.^[32]^ The structure of CPC was confirmed using FTIR and Atomic force microscope (AFM), which acted as the framework for the scaffold (Figure 4h,i). When mixed with CPC, the aMF served as pores and microchannels, promoting better ingrowth of micro‐vessels and micro‐nerves, thereby enhancing the nutrient and oxygen supply.^[33]^ The final product of the 3D‐bioprinted aMF in the CPC scaffold demonstrated an orderly aligned structure, higher shear strength, and elastic modulus than the cancellous bone (Figure 4j,k).

### 2.4 Characterization of the 3D‐bioprinted scaffold

When implanted into the body, a scaffold must first exhibit superior biocompatibility, alongside its effective osteogenic and neurovascular properties. Our CPC‐aMF platform exhibited favorable biocompatibility with surrounding tissues (Figure 4g), and effective protection for the seed cells. SEM images of the CPC scaffold and CPC‐aMF scaffold encapsulating hPDLSCs were taken. Both scaffolds exhibit excellent cell compatibility, evidenced by the even distribution of cells and their elongated morphology. Notably, scaffolds containing alginate microfibers demonstrated cells stretching through the pores following microfiber degradation, forming vessels and nerve ingrowth pathways. However, scaffolds without aMF did not exhibit such a phenomenon (Figure 5a,b). These suggested that the seed cell hPDLSCs had successfully integrated within the scaffold, and CPC‐aMF scaffolds exhibited greater advantages in new nerve and vessel ingrowth than CPC scaffolds (the blank control group).

Consistent with our team's previous findings on the long‐term degradation performance of CPC,[^19^, ^34^] we found that the blank control group degraded only 10%–15% at 1 month, which was not conducive to new bone ingrowth. After incorporating MF, the long‐term degradation performance of the CPC‐MF scaffold was greatly improved (to 30%–40%). In addition, encapsulating the drug (Nell‐1) did not affect the degradation performance of the CPC‐MF platform (Figure 5c). These results suggested that our CPC‐MF platform maintained the stability of the CPC‐based structure as well as the porous and biodegradable MF, degrading neither too early nor too late. This phenomenon was in synchronization with the physiological new tissue formation processes in vivo: it remained stable during the period of connective tissue ingrowth (first 7 days), and provided space for new bone ingrowth during the osteogenic period (at week 4). Interestingly, its early pores structure also provided space for the ingrowth of cells, blood vessels, and nerves.

To achieve a favorable release performance, we chose specifically to encapsulate Nell‐1 in two parts of the scaffold: CPC and aMF. As the CPC was coagulated at the site of the bone defect, the growth factors were released, recruiting endogenous stem cells and related bio‐factors to promote bone formation, ultimately establishing an incipient osteogenic microenvironment.[^35^, ^36^, ^37^] Gradually, long‐lasting growth factors were released from aMF, as it underwent a pre‐designed and two‐stage programmed degradation (Figure 5d). The subsequent growth factors were aimed to promote the proliferation, differentiation, and secretion of essential osteogenic factors by the seeded cells.^[38]^ As shown in the Nell‐1 releasing curve, all groups completed their release at day 12, while the CPC‐only scaffold group reached peak release at day 1–2 (stage one), and the MF‐only group reached peak release at day 6–8 (stage two). Our innovative controlled two‐stage release CPC‐aMF scaffolds outperformed other traditional scaffolds by releasing Nell‐1 at an even keel.

Effective protection for the seed cells was observed under the confocal and light microscopes as well as in the gross appearance (Figure 5e–g). To validate the controlled release of hPDLSCs and Nell‐1, a live/dead assay was conducted. Notably, on day 2, a long fusiform‐shaped cell was observed, indicating the proliferation of the hPDLSCs. At day 14, the seed cells in the scaffold exhibited a well‐proportioned distribution, and the scaffold showed minimal cell toxicity under the confocal microscope (Figure 5h–m). These seed cells served as potential and scattered ossification centers.

### 2.5 3D‐bioprinted aMF in CPC scaffold demonstrated better bone regeneration effects in the rat cranial bone defect model in vivo

**FIGURE 6:**
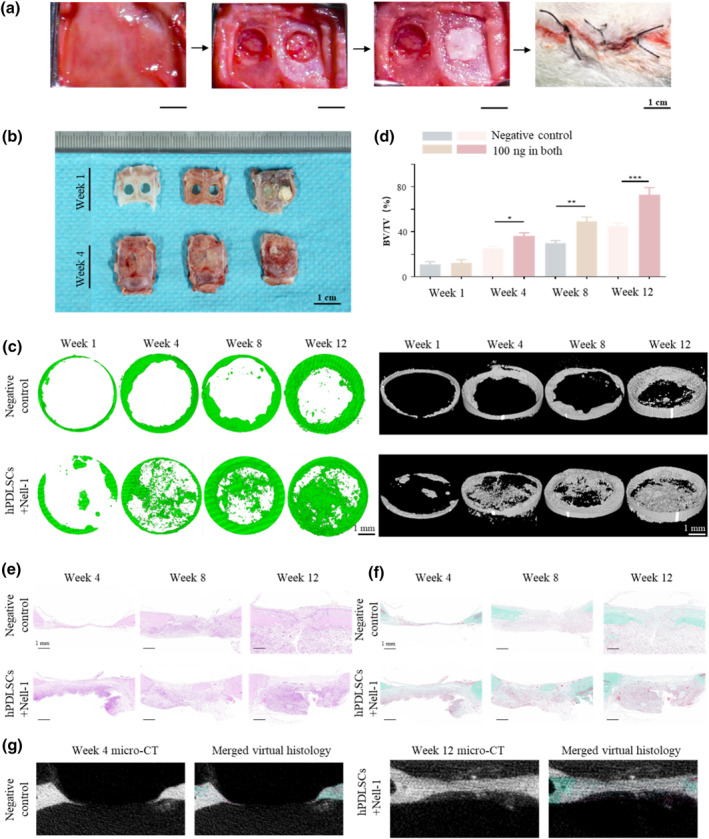
New bone regeneration enhanced by the 3D‐bioprinted aMF in CPC scaffold in vivo. (a) The brief surgical procedure of rat bilateral bone defect modeling. (Scale bar = 1 cm) (b) Photographs of obtained rat bilateral bone specimens at week 1 and 4. (Scale bar = 1 cm) (c) 3D Micro‐CT reconstruction of the bone defect area. (Scale bar = 1 mm) (d) Quantitative analysis of bone volume/tissue volume (BV/TV) of regenerated bone. (e) HE staining of the newly formed bone. (Scale bar = 1 mm) (f) Masson's trichrome staining of the newly formed bone. (Scale bar = 1 mm) (g) Virtual histology of the newly formed bone. (Scale bar = 1 mm) These groups were tested: CPC‐aMF scaffold without hPDLSCs or Nell‐1 as a negative control, CPC‐aMF scaffold with hPDLSCs and 100 ng/mL Nell‐1 in both CPC and aMF (controlled two‐stage release of Nell‐1). All values were presented as the mean ± SD, *p < 0.05, **p < 0.01, and ***p < 0.001 analyzed by one‐way ANOVA (*n* = 4). CPC, calcium phosphate cement.

Rats were used as animal models to evaluate the reparative capacity of the CPC‐aMF scaffold on large cranial bone defects.^[39]^ The construction of the CPC‐aMF two‐stage releasing scaffold involved the combination of CPC and 3D‐bioprinted aMF, which contained hydrogels, Nell‐1, and hPDLSCs (Figure 6a). This scaffold not only provided structural support as strong as the cancellous bone, but also facilitated the sustained and controlled release of Nell‐1 and hPDLSCs.

As depicted in Figure 6a, bilateral cranial bone defects were surgically created on the skull. The CPC‐MF scaffold was then implanted into the defects to investigate its potential for bone tissue regeneration. Remarkably, the group treated with Nell‐1 and hPDLSCs exhibited significantly improved bone regeneration through gross appearance at both 1 and 4 weeks compared to the negative control group (left vs. right, Figure 6b). Moreover, Micro‐CT analysis was performed at 4, 8, and 12 weeks post‐transplantation, demonstrating a significantly enhanced healing of the bone in the Nell‐1 and hPDLSCs groups (Figure 6c,d). In addition, the osteogenic effects of the scaffold were evaluated through HE, Masson staining and virtual histology (Figure 6e–g). Masson staining revealed the presence of newly formed bones, which were demonstrated in green. In summary, these findings indicated that the CPC‐aMF scaffold encapsulating hPDLSCs and Nell‐1 exhibited doubled osteogenic effects compared to the negative control group, leading to a superior bone regeneration effect after its transplantation.

### 2.6 Vascularization and innervation in vivo

**FIGURE 7:**
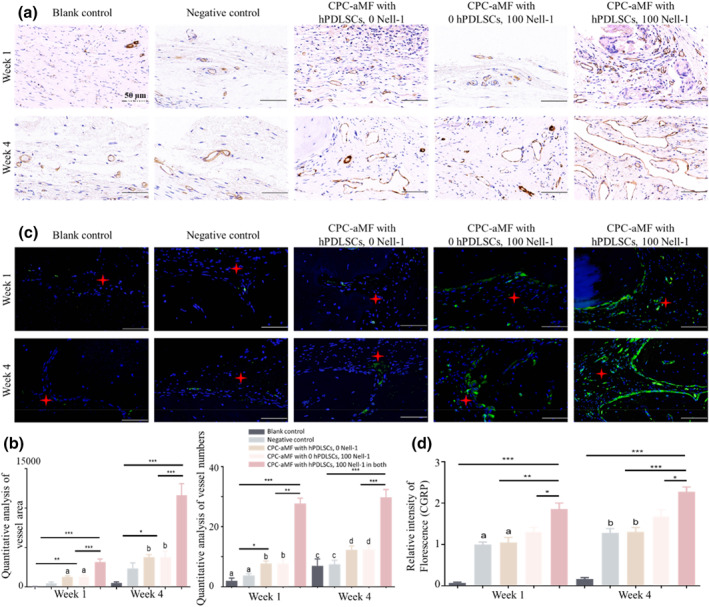
Early vascularization and innervation enhanced by the 3D‐bioprinted aMF in CPC scaffold in vivo. (a) Immunohistochemical staining of CD31. (b) Semi‐quantitation of the area and number of vessels after 1 and 4 weeks according to CD31 expression. Scale bar = 50 μm. (c) Immunohistochemistry staining of calcitonin gene‐related peptide (CGRP) of the bone defect area at week 1 and 4. ★ indicated the medial edge of the bone defect area. (d) Semi‐quantitative analysis of immunofluorescence staining of CGRP. Scale bar = 50 μm. These groups were tested: ①CPC scaffold without hPDLSCs encapsulated, and with 0 Nell‐1 as blank control, ②CPC‐aMF scaffold without hPDLSCs encapsulated, and with 0 Nell‐1 as negative control, ③CPC‐aMF scaffold without hPDLSCs encapsulated, but with 100 ng/mL Nell‐1, ④CPC‐aMF scaffold with hPDLSCs encapsulated, but with 0 Nell‐1, ⑤CPC‐aMF scaffold with hPDLSCs encapsulated, and with 100 ng/mL Nell‐1 in both (controlled two‐stage release of Nell‐1). Scale bar = 50 μm. All values were presented as the mean ± SD, *p < 0.05, **p < 0.01, and ***p < 0.001 analyzed by one‐way ANOVA (*n* = 4). Values with dissimilar letters were significantly different from each other (*p* < 0.05). CPC, calcium phosphate cement.

The hallmark of large bone defect regeneration is now considered to be the ingrowth of capillary‐like vessels.[^40^, ^41^, ^42^, ^43^] Compared to the negative control group, the CPC‐aMF scaffold loaded with hPDLSCs and Nell‐1 showed better promotion of tube formation (Figure 5n,o). In addition, resembling the ingrown vascular networks in vivo, the hPDLSCs and Nell‐1 group showed significantly greater formation of nodes, segments, and total length of micro‐vessels than the blank control group (CPC with 0 hPDLSCs, 0 Nell‐1 blank), and the negative control group (CPC‐aMF with 0 hPDLSCs, 0 Nell‐1 control) (Figure 7a,b). However, there was no significant difference between the blank control group and the negative control group.

These results showed that new vasculature was increased by 3‐fold in vivo with Nell‐1 and hPDLSCs delivery compared to the CPC‐aMF scaffold without Nell‐1 and seed cells. Compared to our previous study,^[44]^ when the blank control group was set as the CPC scaffold only, the blood vessel density was approximately 10% of the whole area. In this study, we set the negative control group as the CPC‐aMF scaffold. The neo‐vessel density was 22%, which was 2‐fold greater than that of the blank control group. We can assume that vascular tissue growth was 6‐fold greater in vivo with Nell‐1 and hPDLSC delivery than that of the blank control group. These results indicated much stronger angiogenesis effects than other currently available scaffolds.

The peripheral nervous system, specifically the sympathetic, parasympathetic, and sensory nerves, plays a crucial role in regulating bone remodeling.^[45]^ In recent years, there has been increasing recognition of the importance of innervation in supplying and modulating bone homeostasis and regeneration.^[46]^ In fact, innervation is often regarded as the final and significant stage of osteogenesis. The development of sensory and autonomic nerves is closely associated with their satellite vascular system in a process known as neuro‐vascularization. They both transverse into the medullary cavity at the end of intramembranous ossification.^[47]^

Pre‐vascularization and advanced angiogenesis prior to osteogenesis are considered hallmarks of bone regeneration. Unfortunately, not much attention has been paid to the importance of innervation in the initial stages. Early innervation, along with the secretion of nerve‐specific bioactive factors,^[48]^ a pivotal event in the early bone healing process. Similar to pre‐vascularization, the rate at which natural nerve formation occurs is much slower than its retraction. The inherent instability of self‐assembled innervation fails to provide an adequate supply of growth factors. Nell‐1, a neural growth factor that is encapsulated and then released from the CPC‐aMF scaffold in two stages, shows great potential in promoting neuro‐vascularized bone regeneration.

The scaffold exhibited superior ability in promoting nerve ingrowth when compared to the negative control group at the early stage. This was evidenced by the enhanced supply of endogenous growth factors and improved integration with bone tissue. Innervation was tested by immunofluorescent staining of CGRP and β3‐Tubulin at weeks 1 and 4. Innervation markers, CGRP (Figure 7c,d) and β3‐Tubulin (Supporting Information S1: Figure S4A,B), first appeared at the edge of the defect as early as week 1, and gradually increased in intensity over time by week 4. These results indicated that nerve tissue growth appeared as early as week 1 and was approximately 2‐fold greater in vivo with Nell‐1 and hPDLSC delivery than the negative control group (CPC‐aMF with 0 hPDLSCs, 0 Nell‐1 negative control). In addition, 20‐fold greater nerve tissue growth was found in vivo with Nell‐1 and hPDLSC delivery than in the blank control group (CPC with 0 hPDLSCs, 0 Nell‐1 blank control).

## 3 DISCUSSION

The present study demonstrates a groundbreaking advancement in bone tissue engineering through the development of a 3D‐bioprinted CPC‐aMF scaffold system incorporating hPDLSCs and featuring a precisely controlled dual‐phase Nell‐1 release mechanism. By incorporating this dual‐phase Nell‐1 delivery, the approach actively promotes the engineering of the “nerve‐bone” axis, successfully addressing the critical challenge of achieving synchronized osteogenesis, angiogenesis, and innervation in large bone defects. The scaffold's design rationale stems from growing recognition that functional bone regeneration requires more than mere mineralized tissue formation, it demands the coordinated development of neural and vascular nanofiber networks. These networks must be biologically and functionally integrated with the osseous component. Our results provide compelling evidence that Nell‐1 serves as a master regulator of this tri‐tissue regeneration process, with its spatiotemporal delivery through our hybrid scaffold creating an optimal microenvironment for complete tissue restoration.

The mechanistic insights gained from this study significantly advanced our understanding of neuro‐osseous interactions. The demonstrated ability of Nell‐1 to activate the CYFIP1‐MAPK‐ERK pathway and subsequently upregulate Runx2 expression established a concrete molecular link between neural signaling and osteogenic differentiation. This finding was particularly noteworthy as it explained how neural elements could directly influence bone formation at the genetic level. Furthermore, the observed 2‐fold increase in CGRP production provided strong evidence for the establishment of a positive feedback loop between nerve ingrowth and bone formation. Nell‐1 served as the central mediator of this reciprocal relationship. In this feedback loop, Nell‐1 was identified as a direct neurotrophic factor. Nell‐1 (Neural epidermal growth factor‐like 1), first discovered in craniosynostosis, could directly promote nerve ingrowth. This direct neurotrophic effect is further amplified by positive feedback once the osteogenic microenvironment is improved. The pharmacological modulation experiments using forskolin and rapamycin confirmed the critical role of the CYFIP1 pathway in mediating Nell‐1's osteogenic effects. These findings offer potential targets for future therapeutic interventions. In summary, within this nerve‐bone positive feedback loop, Nell‐1 produced the most significant osteogenic effects at a concentration of 100 ng/mL.

From a translational perspective, the quantitative improvements achieved by our scaffold system represent a substantial leap forward in regenerative capacity. The 2‐fold enhancement in both bone formation and innervation, coupled with the 3‐fold increase in vascularization, demonstrates the system's ability to overcome the limitations of conventional approaches that focus solely on osteogenesis. Particularly noteworthy is the temporal coordination of these regenerative processes, with early‐stage vascular and neural ingrowth (week 1) preceding and enabling subsequent robust bone formation (week 12). This recapitulation of developmental sequences suggests that our scaffold successfully mimics natural healing cascades, a feature that likely underlies its superior performance. The 6‐fold improvement in vascular network formation compared to CPC‐only scaffolds highlights the critical importance of incorporating both structural (aMF microchannels) and biological (Nell‐1/hPDLSCs) components for optimal tissue regeneration.

The clinical implications of this technology are substantial, particularly for complex bone defects resulting from trauma, tumor resection, or degenerative conditions. The scaffold's ability to simultaneously address the mechanical and biological requirements of large defect repair ‐ through CPC's structural support and aMF's guidance of neurovascular ingrowth ‐ represents a significant advance over current clinical options. It is also crucial to synchronize scaffold degradation and new tissue formation. Our CPC‐MF platform achieved this balance. Its high porosity mimics cancellous bone, providing initial mechanical properties comparable to cancellous bone and allowing vessel and nerve ingrowth. As the MF degrades faster, they create micro‐pores in the CPC‐MF platform. New vessels, nerves, and initial connective tissue grow into these pores, partially replacing the MF structure and thereby helping to maintain the mechanical integrity of the platform. Meanwhile, subsequent controlled degradation creates sufficient space for new bone ingrowth without premature collapse of structural support. Moreover, the use of hPDLSCs offers practical advantages for potential clinical translation, given their relative ease of harvest and strong neuro‐osteogenic potential. The staged release system's capacity to provide both immediate and sustained biological signaling further enhances its therapeutic potential, as it mirrors the dynamic requirements of the healing process. Several important considerations emerge from this work that warrant further investigation. First, while our study has elucidated key aspects of the “nerve‐bone” axis through the CYFIP1‐Runx2‐CGRP pathway, the complete signaling network governing neuro‐osseous interactions remains to be fully mapped. Second, the potential differences in regenerative outcomes between various anatomical sites (e.g., cranial vs. alveolar bone) suggest that scaffold design parameters may need to be optimized for specific clinical applications.^[49]^ Third, the long‐term functional integration of the regenerated neurovascular networks with host tissues requires comprehensive evaluation in large animal models. Finally, the translation of this technology to human‐scale defects will necessitate careful consideration of manufacturing scalability, quality control, and regulatory pathways for this combination product.

This study establishes three transformative concepts in bone tissue engineering: (1) nerve ingrowth is a prerequisite rather than a byproduct of optimal bone regeneration, (2) growth factor delivery must synchronize with multi‐tissue temporal dynamics for functional outcomes, and (3) scaffolds should actively orchestrate neurovascular‐osseous crosstalk rather than passively support osteogenesis. By integrating materials science with developmental biology, we developed a platform that achieves true functional restoration, not just structural repair, through dynamic microenvironmental control. These advances redefine regenerative medicine paradigms, offering a blueprint for neuro‐vascularized tissues (muscle, tendon, osteochondral interfaces) beyond bone. As the field progresses, the core principles demonstrated here—multi‐tissue coordination, developmental sequence recapitulation, and active neuro‐osseous modulation—will guide next‐generation solutions. Future work must now translate these insights into clinical therapies for complex defects while further elucidating the fundamental biology of neurovascular‐osseous interactions.

## 4 CONCLUSIONS

In this study, we present a 3D‐bioprinted CPC‐aMF scaffold with dual‐phase Nell‐1 release and hPDLSCs encapsulation that actively modulates the “nerve‐bone” axis, achieving 2‐fold osteogenesis, 3‐fold angiogenesis, and 3‐fold innervation in rat cranial defects. Notably, the scaffold induced a remarkable 4‐fold increase in new bone formation compared to blank controls, demonstrating its superior regenerative capacity. Mechanistic studies reveal that Nell‐1 mediates neuro‐osseous crosstalk via CYFIP1/PRRC2A signaling, demonstrating for the first time the mechanism by which neural pathways directly enhance bone regeneration. Building on these findings, the scaffold's integrated design combines CPC's mechanical stability, aMF's neurovascular guidance, and the spatiotemporal Nell‐1 delivery to overcome current limitations in functional bone repair. This platform represents a paradigm shift in bone tissue engineering by simultaneously addressing osteogenic, angiogenic, and neurogenic requirements. With demonstrated in vivo efficacy in critical‐sized defects, this technology shows strong potential for clinical translation in complex craniofacial and load‐bearing bone reconstruction, while opening new avenues for studying nerve‐bone interactions in regenerative medicine.

## 5 MATERIALS AND METHODS

### 5.1 Cell culture

HPDLSCs were harvested and cultured as described previously,^[32]^ and this experiment was approved by The Ethics Committee of Beijing Stomatological Hospital affiliated to Capital Medical University, China. HPDLSCs were used at passage 3‐5 for the following experiments. Nell‐1(RND, USA) was added to the medium at concentrations of 0 ng/mL, 50 ng/mL, and 100 ng/mL. Flow cytometry was carried out to characterize the isolated hPDLSCs. A cell suspension containing 1 × 10^6^ hPDLSCs and antibodies against CD90, CD105, CD34, CD45, CD106, and STRO‐1 (Thermo Fisher Scientific, Waltham, MA, USA) was analyzed through flow cytometry (Accuri C6 Flow Cytometer, BD Bioscience, San Jose, CA, USA).

### 5.2 In vitro osteogenic experiments

7‐day, 14‐day, and 21‐day‐old hPDLSCs were stimulated under the effect of Nell‐1, and a control group was also included for comparison. To evaluate the osteogenic effects and quantify the mineral synthesized by hPDLSCs, the samples were stained with ALP and ARS. ALP and ARS were analyzed later using a light microscope for visualization and semi‐quantification. Real‐time polymerase chain reaction (RT‐PCR) analysis was performed to examine the expression of osteogenic markers (ALP) (Supporting Information S1: Table S2) using the PrimeScriptTM Master Mix system (TaKaRa, Japan). Total proteins were extracted using RIPA buffer, and Western blotting was conducted following the manufacturer's instructions to compare the levels of these osteogenic markers.

### 5.3 Western blot

The human CYFIP1 (NM_014608.6) was amplified from a cDNA library using primers as detailed in Supporting Information S1: Table S2. The GV657 vector and the CYFIP1 gene sequence were digested with MSC restriction enzymes (BamHI and KpnI) and subsequently subjected to complete cloning via the in‐fusion recombination technique. Additionally, a plasmid vector engineered to express shRNA targeting the CYFIP1 gene sequence (as detailed in Supporting Information S1: Table S2) was synthesized and cloned into the GV493 vector at MSC restriction sites (AgeI and EcoRI). The shRNA‐CYFIP1 plasmid was purified from *Escherichia coli* using the Endo‐free plasmid Mega kit. Finally, the CYFIP1 overexpression and knockdown plasmids were applied to hPDLSCs with 100 ng/mL Nell‐1.

Western blot analysis was then conducted to assess the expression of CYFIP1 in hPDLSCs. The protein levels of Runx2 were examined across the following experimental groups: Control, Agonist, Inhibitor, Overexpression, and Knockdown (*n* = 4).

The groups subjected to protein expression analysis included:

1. Nell‐1 (100 ng/mL Nell‐1)
2. Agonist (100 ng/mL Nell‐1 with 100 μM CYFIP1‐cAMP agonist Forskolin)
3. Inhibitor (100 ng/mL Nell‐1 with 100 μM CYFIP1‐mTOR inhibitor rapamycin)
4. Overexpress (100 ng/mL Nell‐1 with shRNA for CYFIP1 gene overexpression)
5. Knockdown (100 ng/mL Nell‐1 with shRNA for CYFIP1 gene knockdown)

Proteins were extracted from the cellular samples, and the expression of Runx2 was quantified relative to GAPDH, which served as the loading control.

### 5.4 RNA sequencing analysis

Total RNA was isolated using the TRIzon Reagent kit (Cowin Biotech, China). Subsequent RNA sequencing was performed, filtered, and further subjected to the standard protocols. Volcano plot and heatmap were drawn to depict the differential gene expression in the samples. KEGG and GO enrichment analysis was conducted using Phyper. Additionally, CYFIP1 and PRRC2A networks were analyzed using STRING and corrected with a rigorous threshold determined by the *Q* value.

To assess the angiogenic effects, a tube formation assay was conducted. A 96‐well plate was precoated with 50 μL of Matrigel per well, and HUVECs, hPDLSCs, and Nell‐1 at concentrations of 0 ng/mL or 100 ng/mL were inoculated into the wells. The formation of tube‐like structures was observed after 6 h using an inverted light microscope. Immunofluorescence staining of the angiogenic marker CD31 was also performed to compare vascularization using confocal microscopy. Further quantification of the results was carried out using ImageJ (Angiogenesis Analyzer).

### 5.5 Fabrication of 3D‐bioprinted aMF in CPC scaffold

The bioink suspension (Supporting Information S1: Table S1) containing hPDLSCs (a total of 1 × 10^6 cells), Nell‐1, and alginate phosphate was loaded into the needle tube of the printer (Allevi, USA). The FRESH bath containing an additional 0.1% CaCl_2_ was fabricated to support the structure of the scaffold. The printing chamber was maintained at a constant temperature of 25°C. The detailed printing process was demonstrated in Movie S1. During printing, the needle diameter, speed, and extrusion pressure were kept constant at 27 G, 10 mm/s, and 1.5–1.8 kPa, respectively, as previously reported.^[31]^ The diameter of the microfibers ranged from 150 to 200 μm. After 3D printing, the hydrogel microfibers were crosslinked in a 1% CaCl_2_ warm bath at 37°C, and extra dissipated gelatin was carefully removed from the dish. Combined, CPC‐alginate microfiber scaffolds were formed by 3D‐bioprinted alginate microfibers, tetra calcium phosphate, dicalcium phosphate‐anhydrous, and Chitosan solution with 100 ng/mL of Nell‐1. Nell‐1 was encapsulated both in the 3D‐printing bioink with hPDLSCs (0 ng/mL, 50 ng/mL, and 100 ng/mL) and in the CPC scaffold (0 ng/mL, 100 ng/mL) for two‐stage release.

### 5.6 Characterization of the scaffolds

The CPC scaffolds were then tested with Fourier‐transform infrared spectroscopy (FTIR) and AFM. In order to evaluate the mechanical properties and to demonstrate the advantages of two‐stage releasing scaffolds, these groups were tested:

1. Blank control (CPC scaffold only)
2. Negative control (CPC‐aMF with 0 Nell‐1 control)
3. CPC‐aMF with 100 ng/mL Nell‐1 in CPC
4. CPC‐aMF with 100 ng/mL Nell‐1 in aMF
5. CPC‐aMF with 100 ng/mL Nell‐1 in both
6. Cancellous bone (bone control)

In addition, these scaffolds underwent biomechanical evaluation through cyclic loading with compressive strain applied up to 30% of their initial thickness over 10 cycles. The loading rate was 0.5 mm/min. The resulting fatigue compression loading and unloading curves were analyzed accordingly.

The compatibility of the CPC scaffolds with and without microfibers and their pre‐vascularization ability were demonstrated with Scanning electron microscopy (SEM) after 21 days of incubation. The mechanical properties, flexural strength, elastic Modulus, and cyclic loading of the CPC‐aMF scaffolds were tested by a universal testing machine.

In Vitro Degradation Study. Fully cured scaffolds were immersed in PBS buffer and maintained a sample‐to‐buffer ratio of 0.1 g/mL.^[50]^ At each time point (from day 0 to day 28), the scaffolds were retrieved, rinsed and dried. The dried scaffolds were then weighed to obtain the mass and to calculate the degradation rate. Furthermore, the concentration of Nell‐1 released from the two‐stage scaffolds was measured using a Nell‐1 ELISA kit from day 0 to day 12. In addition, the changes in pH levels in PBS were monitored throughout the degradation process of the scaffolds.

The designed degradation rate of the 3D‐bioprinted hydrogel microfibers, and the viability (live/dead assay) of the encapsulated hPDLSCs after printing were then validated. The microfibers were measured after incubating them at 37°C with growth medium for 0, 2, 4, 8, and 14 days under light and confocal microscopes (Leica, Japan), respectively.

### 5.7 Animal experiment

The animal experiment was approved by the Animal Ethical Committee of Beijing Stomatological Hospital affiliated to Capital Medical University, China. Male Sprague‐Dawley rats were used as animal models to evaluate the reparative capacity of the CPC‐aMF scaffold on large cranial bone defects. Different groups of 3D‐bioprinted aMF in CPC scaffolds were then transplanted into the bilateral sides of rat models with cranial bone defects in vivo. After 1, 4, 8, and 12 weeks, the cranial bone tissues were retrieved and examined for osteogenic, angiogenic, and innervation markers through immunofluorescence. Neo‐bone regeneration and neuro‐vascularization were determined through Masson staining and Micro‐CT analysis. SD rats weighing 180–220 g were used in this study for in vivo experimentation. The rats were randomly assigned to five groups (both sides of the cranial bone were used):

1. Blank control (CPC only with 0 hPDLSCs, 0 Nell‐1 blank)
2. Negative control (CPC‐aMF with 0 hPDLSCs, 0 Nell‐1 control)
3. CPC‐aMF with 0 hPDLSCs, 100 ng/mL Nell‐1
4. CPC‐aMF with hPDLSCs, 0 Nell‐1
5. CPC‐aMF with hPDLSCs, 100 ng/mL Nell‐1 in both

After anesthesia and sterilization, the rats' skull was exposed along the mid‐sagittal crest for an adequate surgical field. Created a full‐thickness cylindrical defect (5 mm diameter) on both sides with a ring drill.[^51^, ^52^] The 3D‐bioprinted aMF encapsulating hPDLSCs in the CPC scaffold with two‐stage Nell‐1 release was implanted to match the defect. Saline cooling was employed throughout the procedure to prevent excess heat generated by CPC coagulation. Lastly, the surgical incision of the skull was closed with sutures, and antibiotics were administered.

After 1, 4, 8, and 12 weeks, specimens of the cranial bone tissue were harvested and sagittally cut through the center. 3D Micro‐CT, HE, Masson staining and virtual histology were performed following the standard protocols. Histological, immunohistochemical, and immunofluorescence staining for osteogenic markers (Runx2 and OCN), angiogenic markers (CD31 and α‐SMA), and innervation markers (CGRP and β3‐Tubulin) were also conducted using standard protocols. The stained images were captured, semi‐quantified, and analyzed using a light microscope and ImageJ.

### 5.8 Statistical analysis

The data obtained were statistically analyzed using SPSS software (version 20.0) and are presented as mean ± standard deviation (SD). All experiments were performed with three replicates in vitro and four replicates in vivo, respectively. Comparisons among different groups were analyzed by *t*‐tests and one‐way ANOVA. The level of significance was set at *p < 0.05, **p < 0.01, and ***p < 0.001.

## AUTHOR CONTRIBUTIONS

Minjia Zhu made substantial contributions to the conception and methodology visualization and wrote the original draft; Minjia Zhu and Kan Yu made contributions to formal analysis; Minjia Zhu, Kan Yu, Zixiang Dai, Le Xiao, Qinrou Zhang, Xinyi Li, Jingyi Li, Zihan Jia, Qingchen Qiao and Zeqing Zhao made contributions to investigation; Yuxing Bai made contributions to supervision; Ke Zhang made contributions to funding acquisition, resources and supervision and reviewed and edited the text.

## CONFLICT OF INTEREST STATEMENT

The authors declare no conflicts of interest.

## ETHICS STATEMENT

This experiment was approved by The Ethics Committee of Beijing Stomatological Hospital affiliated to Capital Medical University, China. The animal experiment was approved by the Animal Ethical Committee of Beijing Stomatological Hospital affiliated to Capital Medical University, China.

## CONSENT FOR PUBLICATION

All authors contributed to the article and approved the final manuscript.

## Supporting information

Supporting Information S1

Video S1

## Data Availability

The data that support the findings of this study are available from the corresponding author upon reasonable request.
