# Supplementary material for: Innovative 3D‐bioprinted microfibers in calcium phosphate cement platform with Nell‐1 to activate nerve‐bone axis for synergistic bone, vasculature, and nanofibrous nerve regeneration
Source: Smart Mol. 2026 Jul 15:e70078. Online ahead of print. doi: 10.1002/smo2.70078 (PMC13398944; doi:10.1002/smo2.70078)

Supporting Information

Innovative 3D-bioprinted microfibers in CPC platform with Nell-1 to activate nerve-bone axis for synergistic bone, vasculature, and nanofibrous nerve regeneration

Minjia Zhu, Xinyi Li, Jingyi Li, Kan Yu, Zixiang Dai, Le Xiao, Qinrou Zhang, Zihan Jia, Qingchen Qiao, Zeqing Zhao, and Ke Zhang*, Yuxing Bai*

**This PDF file includes:**

Figs. S1 to S4

Tables S1 to S2

Movies S1

**Other Supplementary Materials for this manuscript include the following:**

Movies S1

**Fig. S1.**


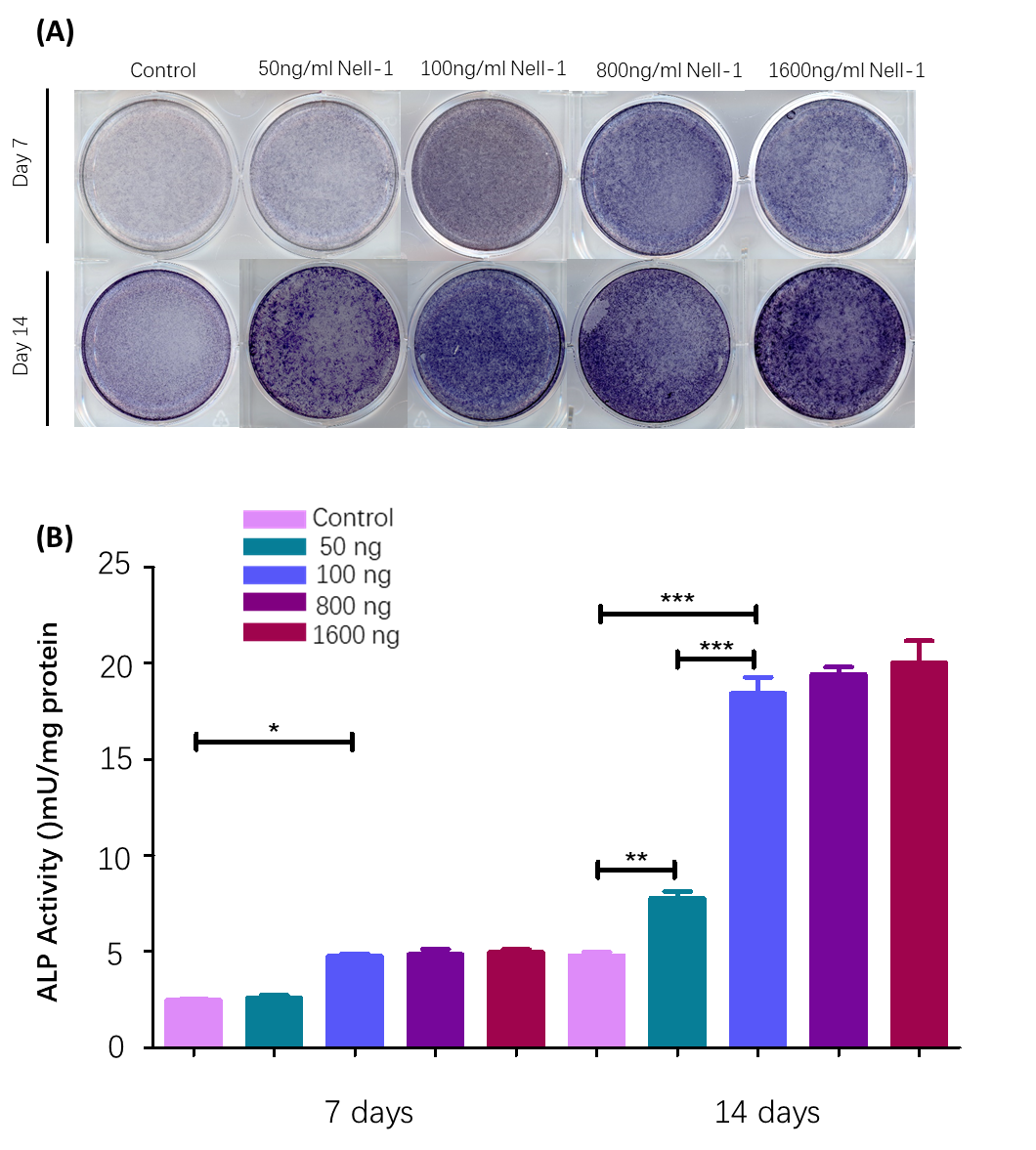


**Fig. S1. ALP staining of hPDLSCs with different concentrations of Nell-1.** (**A**) ALP staining of hPDLSCs with 0ng, 50ng, 100ng, 800ng and 1600ng Nell-1 after 7 and 14 days of culture. (**B**) Semi-quantification of ALP activity of hPDLSCs with 0ng, 50ng, 100ng, 800ng and 1600ng Nell-1 after 7 and 14 days of culture. All values were presented as the mean ± SD, ^*^*P* < 0.05, ^**^*P* < 0.01, and ^***^*P* < 0.001 analyzed by one-way ANOVA (n = 4).

**Fig. S2.**


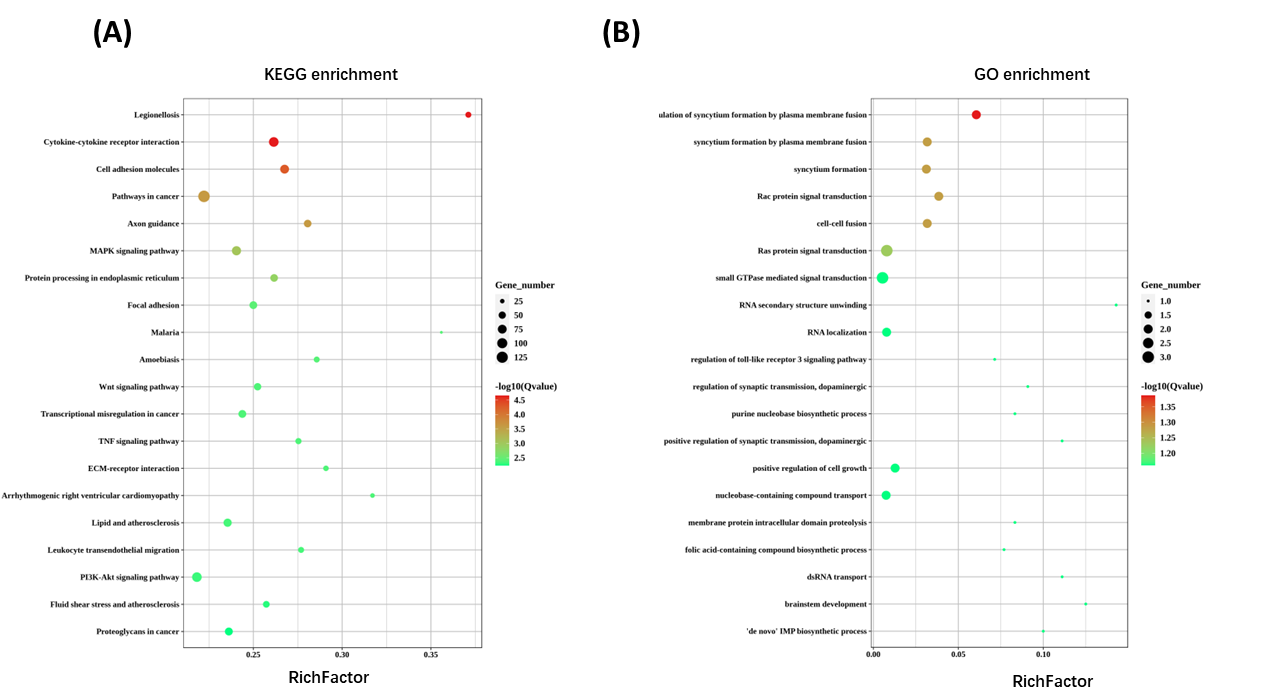


**Fig. S2. Different enrichment analysis for differentially expressed genes.** (**A**) KEGG enrichment analysis for differentially expressed genes. (**B**) GO enrichment analysis for differentially expressed genes.

**Fig. S3.**


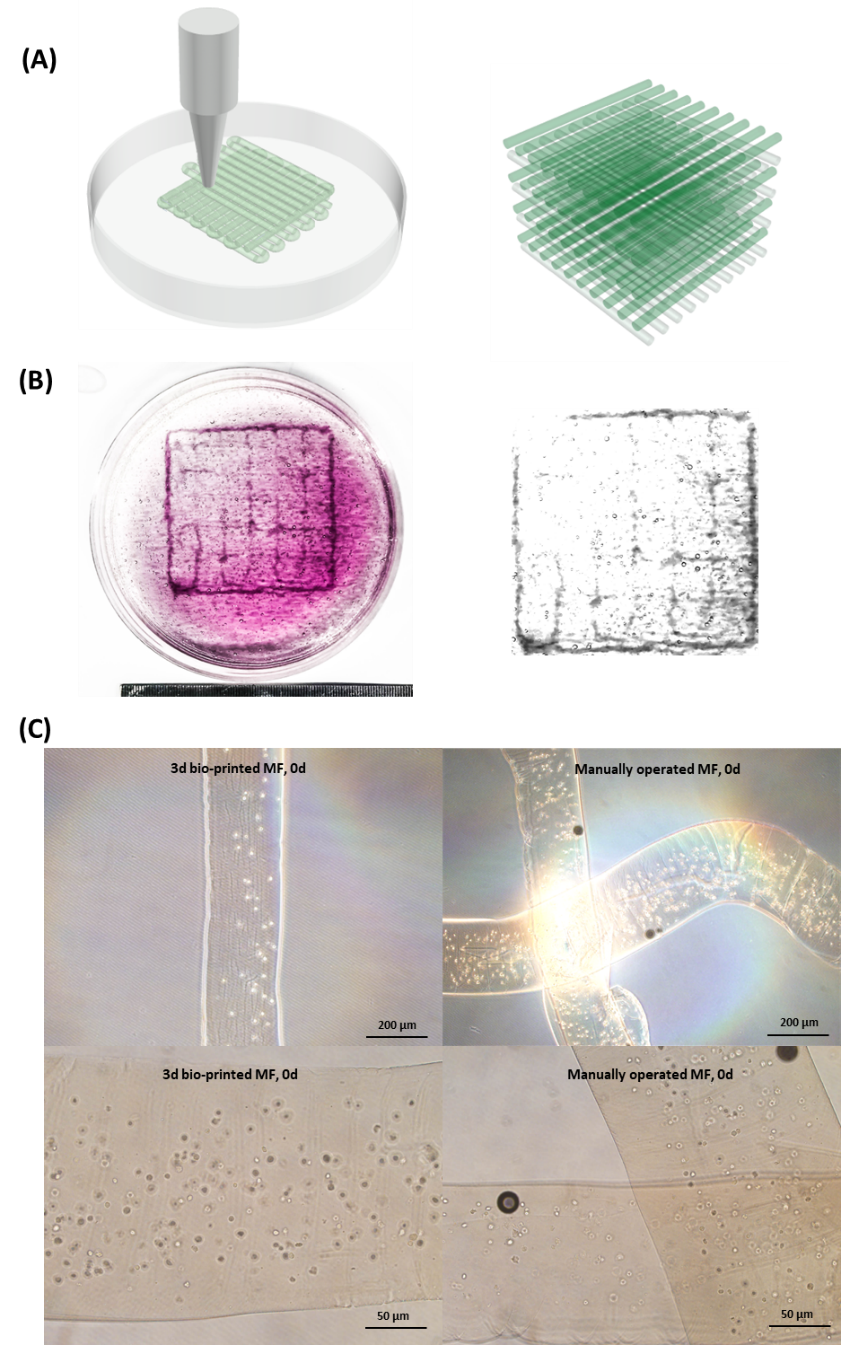


**Fig. S3. 3D bio-printing microfiber MF.** (**A**) A computer-aided design model for 3D bio-printing MF. (**B**) Gross appearance of the 3D bio-printing MF as 27G, 10mm/s, and 1 kPa. Scale bar = 1 cm. (**C**) Light microscopic appearance of the microfiber (encapsulated with hPDLSCs and Nell-1) with and without 3D bio-printing. Scale bar = 200 μm, 50 μm.

**Movie S1. (separate file)**

**
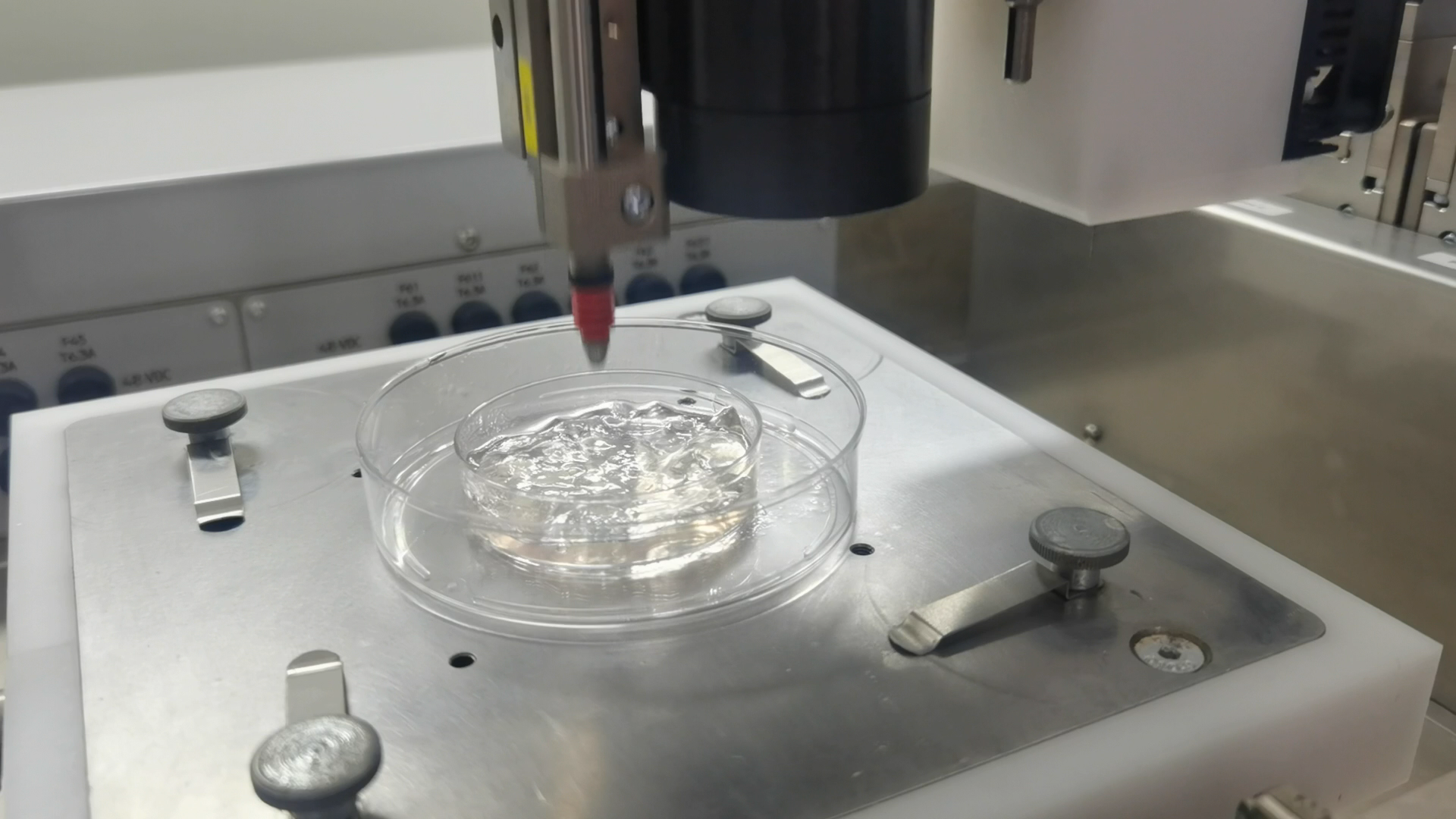
**

**Movie S1. 3D bio-printing microfiber MF.** (**A**) 3D bio-printing microfiber following the FRESH bath strategy.

**Fig. S4.**


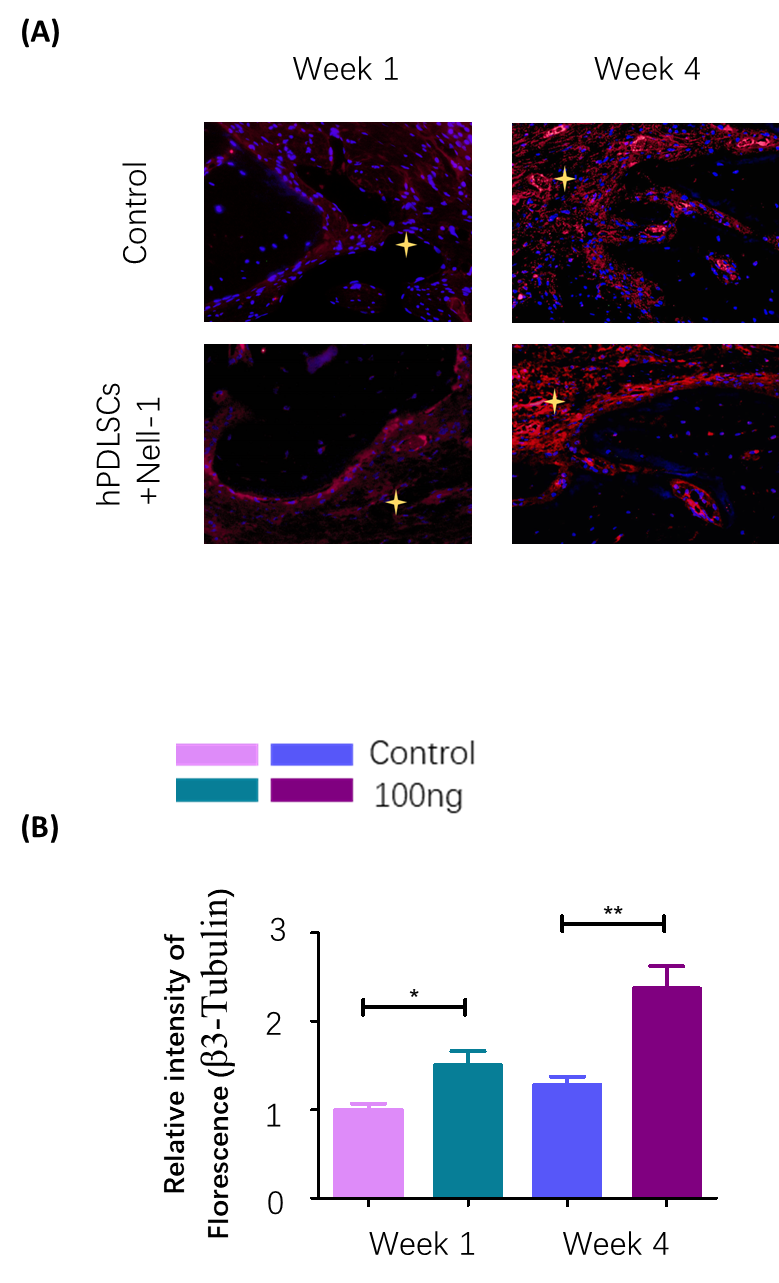


**Fig. S4. Early innervation enhanced by the 3D bio-printing CPC-microfiber scaffold *in vivo*.** (**A**) Immunohistochemistry staining of β3-Tubulin of the bone defect area at week 1 and 4. ★ indicated the medial edge of the bone defect area. (**B**) Semi-quantitative analysis of immunofluorescence staining of β3-Tubulin. All values were presented as the mean ± SD, ^*^*P* < 0.05, ^**^*P* < 0.01, and ^***^*P* < 0.001 analyzed by one-way ANOVA (n = 4).

**Table S1.**

**Table S1. Preparation of the bioink for 3D bio-printing MF.**


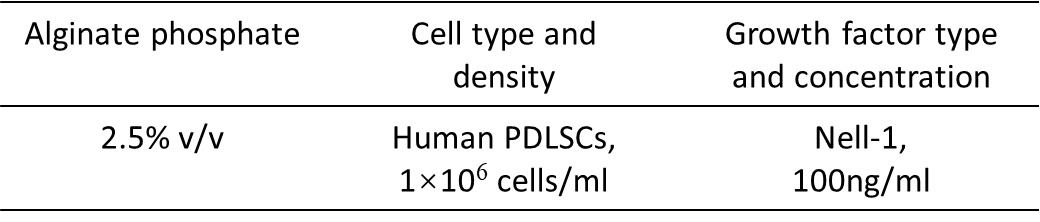


**Table S2.**

**Table S2. Sequences of primers for RT-PCR experiments.** The sense and antisense PCR primers used are listed in Table S2 as follows.


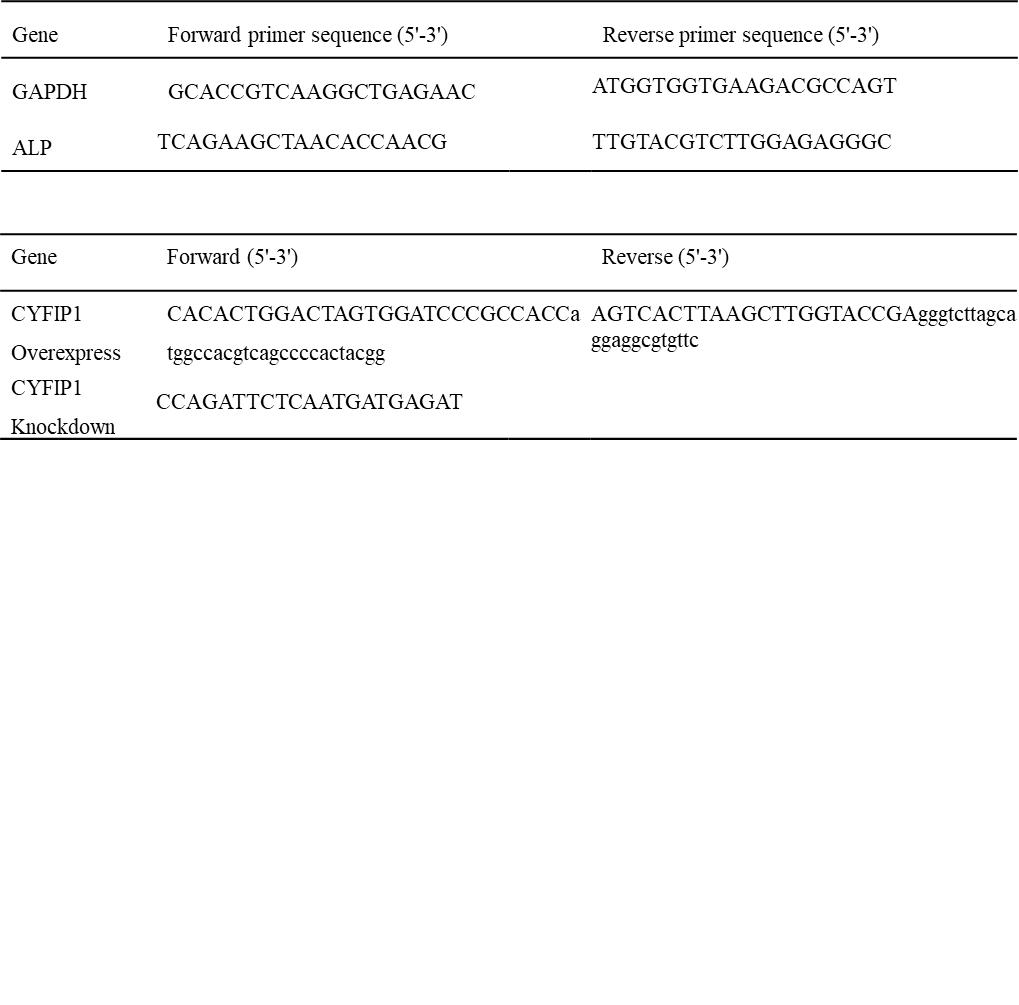

Supplement: Supplementary file 1 — Supporting Information S1 [file SMO2-9999-0-s001.docx]
